# Supplementary material for: Kub3 Deficiency Causes Aberrant Late Embryonic Lung Development in Mice by the FGF Signaling Pathway
Source: Int J Mol Sci. 2022 May 27;23(11):6014. doi: 10.3390/ijms23116014 (PMC9181541; doi:10.3390/ijms23116014)
Supplement: Supplementary file 1 [file ijms-23-06014-s001.zip › ijms-1687725-SI.pdf]

International Journal of
Molecular Sciences

MDPI

Kub3 Deficiency Causes Aberrant Late Embryonic Lung Development in Mice by the FGF Signaling Pathway

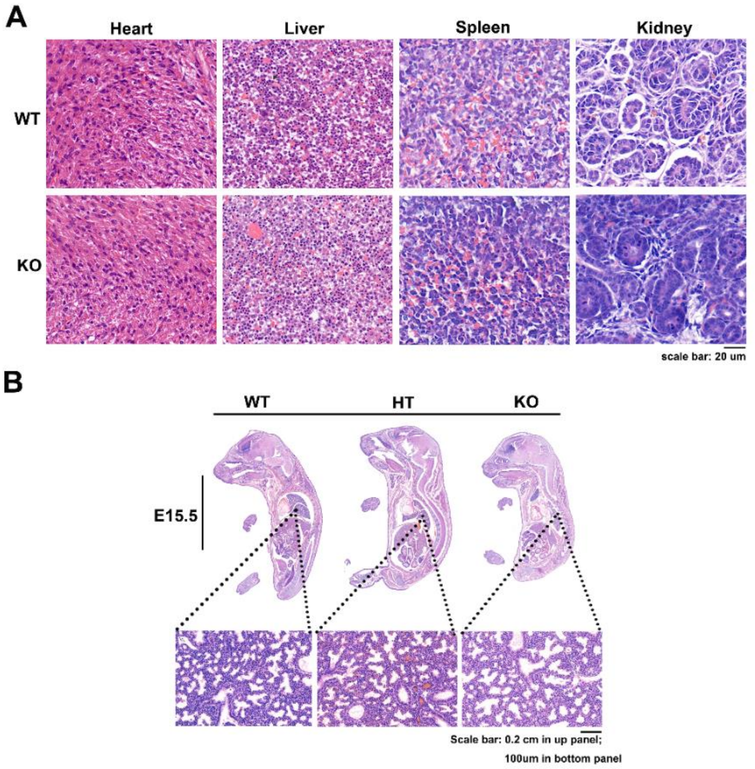

Figure S1. *Kub3* deletion has no obvious effect on morphological structure of other organs at E18.5 and of morphological structure of lung at E15.5. **(A)** Morphological structure of heart, liver, spleen and kidney in E18.5 WT and KO embryo was examined by hematoxylin and eosin (H&E) staining. Scale bar, 20 µm. **(B)** Morphological structure of E15.5 embryos examined by H&E staining. Images of lung tissues are enlarged in bottom panel. Scale bar, 0.1 cm in upper panel; 200 µm in bottom panel.

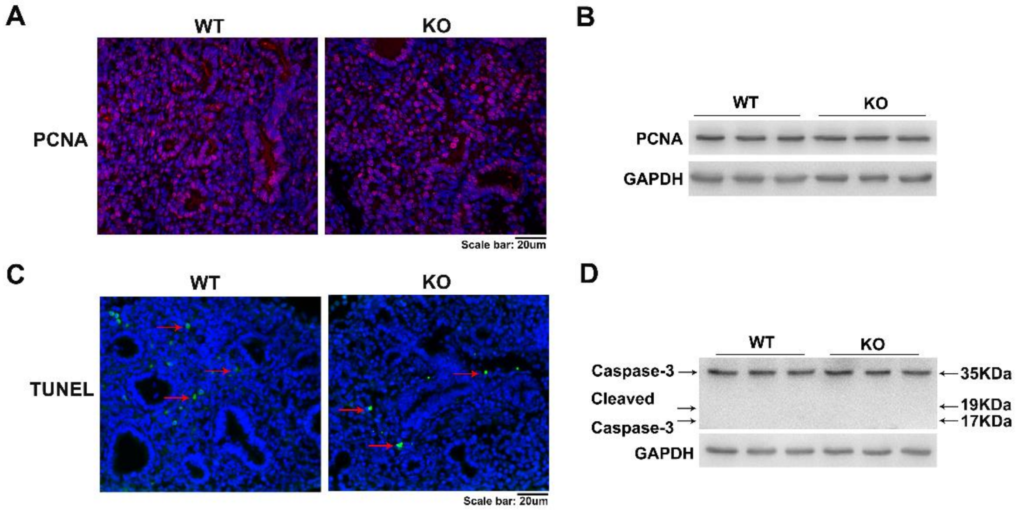

Figure S2. *Kub3* deletion has no effect on lung cell proliferation and cell apoptosis at E15.5. **(A)** Immunostaining of PCNA in lung sections of E15.5 lungs. Nuclei were stained blue with DAPI. Scale bar, 50 µm. **(B)** Western-blot analysis of PCNA protein level in E15.5 lungs. GAPDH is used as loading control. **(C)** Cell apoptosis analysis of cells in E15.5 lungs examined by terminal

deoxynucleotidyl transferase dUTP nick end labeling (TUNEL) assay. Arrows indicate TUNEL-positive nuclei. Scale bar, 20 µm. **(D)** Western-blot analysis of protein levels of caspase-3 and cleaved caspase-3 in KO lung at E15.5. GAPDH is the protein loading control.

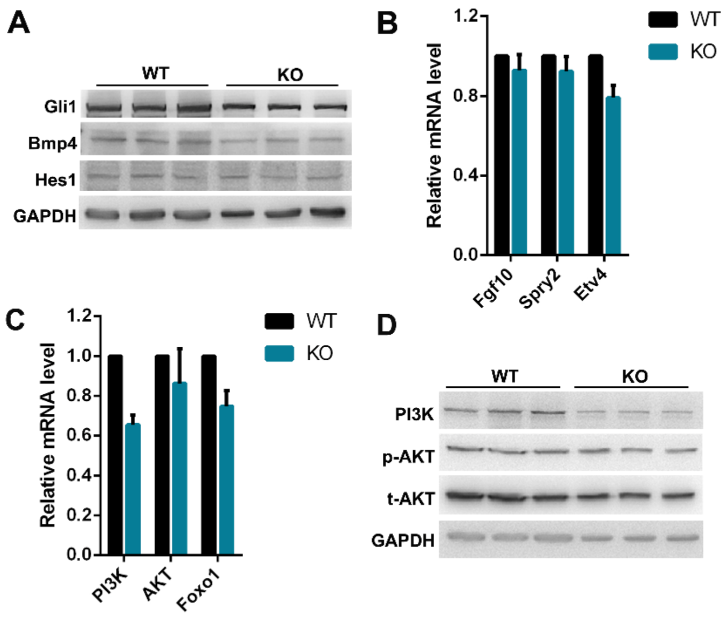

Figure S3. (A) Western-blot analysis of protein levels of marker proteins for Shh, Bmp, and Notch signaling pathway in E18.5 lungs. Protein lysates were subjected to Western-blot and performed with the indicated antibodies. GAPDH was used as a loading control. (**B)** Quantitative RT-PCR analysis of the mRNA levels of marker genes for FGF signaling pathway in E15.5 lungs. The data are derived from three independent experiments performed in triplicate and are normalized to the GAPDH (n=3). **(C)** Quantitative RT-PCR analysis of the mRNA levels of marker genes for PI3K-AKT and PLCγ1 signaling pathway in E18.5 lungs. PI3K and AKT are marker genes for PI3K-AKT pathway, and FOXO1 is the marker gene for PLCγ1 pathway. The data are derived from three independent experiments performed in triplicate and are normalized to the GAPDH (n=3). **(D)** Western-blot analysis of protein levels of marker proteins for PI3K-AKT pathway in the lungs of *Kub3* WT and KO at E18.5. Protein lysates were subjected to Western-blot and performed with the indicated antibodies. GAPDH was used as a loading control.

Table S1. The primers for qPCR.

Gene	Primer sequence (5'-3')
mq-GAPDH-F	ACCACAGTCCATGCCATCAC
mq-GAPDH-R	TCCACCACCCTGTTGCTGTA
mq-Kub3-F	CTCATTCACGCGTTTGACCA
mq-Kub3-R	CAAAACGCAGCCTGAAGAGT
mq-SP-A-F	ACTCCCATTGTTTGCAGAATC
mq-SP-A-R	AAGGGAGAGCCTGGAGAAAG
mq-SP-B-F	GAACTGTGTAGCGCTCAGCC
mq-SP-B-R	CTCTGATCAAGCGGGTTCAA
mq-SP-C-F	ATGAGAAGGCGTTTGAGGTG
mq-SP-C-R	AGCAAAGAGGTCCTGATGGA
mq-SP-D-F	GAGAGCCCCATAGGTCCTG
mq-SP-D-R	GTAGCCCAACAGAGAATGGC
mq-Abca3-F	CTGGCATACCACTTCGCTTT
mq-Abca3-R	TAACGCATGATGGCTTTGTC
mq-Aqp5-F	ATCTCCATAGCCTTTGGCCT
mq-Aqp5-R	TAGAAGATGGCTCGGAGCAG
mq-Pdpn-F	GAGGCTCCAACGAGATCAAG
mq-Pdpn-R	GTCTCCTGTACCTGGGGTCA
mq-Wnt2-F	TCTTGAAACAAGAATGCAAGTGTCA

mq-Wnt2-R	GAGATAGTCGCCTGTTTTCCTGAA
mq-Wnt5a-F	AATCCACGCTAAGGGTTCCT
mq-Wnt5a-R	GAGCCAGACACTCCATGACA
mq-Wnt7b-F	GGATGCCCGTGAGATCAAAA
mq-Wnt7b-R	CACACCGTGACACTTACATTCCA
mq-Axin2-F	CAGCCCTTGTGGTTCAAGCT
mq-Axin2-R	GGTAGATTCCTGATGGCCGATGT
mq-Shh-F	AATCTGCAACGGAAGCGAGG
mq-Shh-R	AACAGCCAGGTGCCAATGTG
mq-Gli1-F	TGCCAGATATGCTTCAGCCA
mq-Gli1-R	TGTGGCGAATAGACAGAGGT
mq-Gli2-F	AGCCCATGACTCTCACCTCCAT
mq-Gli2-R	TCGCTGTTCTGCTTGTTCTGGTT
mq-Gli3-F	AGCAACCAGGAGCCTGAAGTCAT
mq-Gli3-R	GTCTTGAGTAGGCTTTTGTGCAA
mq-Bmp4-F	CGTTACCTCAAGGGAGTCGAGATTG
mq-Bmp4-R	TCTTATTCTTCTTCCTGGACCGCTG
mq-Hes1-F	GGCAGACATTCTGGAAATGA
mq-Hes1-R	TTGATCTGGGTCATGCAGTT
mq-Fgf10-F	GGAGAGAGGAGATTCTTCTTCAC
mq- Fgf10-R	CCGTGGCTAACACACTTCAG
mq-Spry2-F	GGAACAGAGACTGTTAGGACCG
mq-Spry2-R	CCTTGCTCAGTGGCTTAACGTC
mq-Etv4-F	GGCAGAACAGCAGCAGAGC
mq-Etv4-R	CCTCCCTGAGGAGATGTGAA
mq-PI3K-F	GCCCAGGCTTACTACAGAC
mq-PI3K-R	AAGTAGGGAGGCATCTCG
mq-AKT-F	AGTCCCCACTCAACAACTTCT
mq-AKT-R	GAAGGTGCGCTCAATGACTG
mq-Foxo1-F	AACCAGTCCAACTCGACCAC
mq-Foxo1-R	TTCTAGCAGGCTCAGGTTGC
